# Nucleated red blood cells as early predictors of poor neurological outcome after extracorporeal cardiopulmonary resuscitation: a retrospective dual-site cohort study of early laboratory markers

**DOI:** 10.1016/j.resplu.2026.101340

**Published:** 2026-04-24

**Authors:** Julius Valentin Kunz, Mareen Pigorsch, Jens Nee, Lilly Koppelkamm, Teresa Carola Juchem, Roland Körner, Kai-Uwe Eckardt, Philipp Enghard, Jan Matthias Kruse, Abakar Magomedov

**Affiliations:** a Charité – Universitätsmedizin Berlin, Corporate Member of Freie Universität Berlin and Humboldt Universität zu Berlin, Department of Nephrology and Medical Intensive Care, Charitéplatz 1, 10117 Berlin, Germany; b Charité – Universitätsmedizin Berlin, Corporate Member of Freie Universität Berlin, Institute of Biometry and Clinical Epidemiology, Humboldt Universität zu Berlin, Charitéplatz 1, 10117 Berlin, Germany

**Keywords:** ECPR, Cardiac arrest, Circulatory arrest, Neuroprognostication, Nucleated red blood cells, Lactate, pH, Biomarkers

## Abstract

**Background:**

Early neurological prognostication after extracorporeal cardiopulmonary resuscitation (ECPR) remains difficult. Common pre-cannulation markers such as lactate and pH offer limited accuracy for predicting neurological outcome. Nucleated red blood cells (NRBCs) increase under hypoxemia, yet their value as early predictors of adverse neurological outcome after ECPR has not been evaluated.

**Methods:**

We performed a retrospective dual-site cohort study of adult patients who underwent ECPR for refractory circulatory arrest at Charité–Universitätsmedizin Berlin (2020–2024). Laboratory parameters obtained within six hours after admission were analyzed regarding their association with poor neurological outcome at discharge, defined as Cerebral Performance Category 3–5 or death before neuroprognostic assessment. Group differences were assessed using Cliff’s Δ, and discriminative performance was evaluated by receiver operating characteristic analysis. Poor neurological outcome served as the positive condition, with rule-in (≥90% specificity) and rule-out (≥90% sensitivity) thresholds.

**Results:**

Among 197 patients (median age 55 years [IQR 46–61], 86.0% male), 38 (19.3%) had a good outcome and 159 (80.7%) a poor outcome. NRBCs/100 white blood cells (WBCs) showed the strongest association with outcome: patients with poor outcome had higher values (0.37 *n*/100 WBCs [0.18–0.72] vs. 0.19 *n*/100 WBCs [0.07–0.45]; Cliff’s Δ = 0.39, 95% CI 0.15–0.58). The area under the curve (AUC) was 0.70 [95% CI 0.59–0.80]. A rule-in threshold ≥0.54 *n*/100 WBCs (sensitivity 33%, specificity 93%) identified 42 true and only 2 false poor-outcome cases. Initial lactate (AUC 0.69 [0.59–0.80]) and pH (AUC 0.67 [0.55–0.79]) produced rule-in thresholds ≥18.4 mmol/L and ≤6.72, identifying 38 true vs. 3 false and 24 true vs. 2 false poor-outcome patients. WBCs, follow-up lactate, and phosphate were also associated with poor outcome. At rule-out thresholds, all markers performed poorly, with specificities of only 16–35% and frequent false-good classifications, underscoring the greater reliability of rule-in approaches.

**Conclusions:**

NRBCs/100 WBCs measured within six hours after admission showed the best discriminatory ability for poor neurological outcome, outperforming lactate, pH, and other routine laboratory markers. NRBCs may represent a promising early indicator requiring prospective validation.

## Highlights

• NRBC/100 WBC is the strongest early laboratory predictor of poor outcome after ECPR.
• Early laboratory markers enable reliable rule-in but not rule-out prognostication.
• Pre-cannulation lactate outperforms pH for early neurological prognostication.

## Introduction

The use of extracorporeal cardiopulmonary resuscitation (ECPR) for refractory circulatory arrest has expanded substantially over the past decade as dedicated programs have matured within specialized circulatory arrest centers. Evidence from recent studies suggests that ECPR is associated with improved survival and neurological outcomes compared with conventional cardiopulmonary resuscitation (CPR).^1^, ^2^, ^3^

Current guidelines emphasize that reliable neuroprognostication after ECPR or conventional CPR is only recommended at ≥72 h after resuscitation with a multimodal assessment.^4^ Identifying earlier prognostic indicators remains an ongoing challenge.

Among laboratory findings, pH and lactate at the time of cannulation have been most consistently studied in ECPR cohorts. Lower lactate and higher pH values at initiation have repeatedly been reported in patients with favorable outcomes compared to those with poor outcomes. At the time of ECPR initiation, metabolic parameters such as lactate and pH reflect the extent and duration of preceding circulatory failure. Initial lactate concentrations above 14.0 and 15.1 mmol/L respectively have been consistently linked to markedly reduced survival and poor neurological recovery, whereas lower values are associated with better prognosis.^5^, ^6^

Furthermore, dynamic changes appear relevant: multicentre data demonstrated that both initial lactate and its early reduction within 24 h correlate with long-term outcome,^6^ while a lactate clearance above 65% within the first six hours after ECPR was associated with markedly higher survival in one cohort.^7^

Plasma pH at cannulation likewise demonstrated prognostic relevance. Both an initial pH above 7.0 and the presence of a shockable rhythm were independently associated with favorable neurological outcomes.^8^ Furthermore, a meta-analysis identified low pH as one of the most consistent predictors of mortality among patients undergoing ECPR after out-of-hospital circulatory arrest.^9^

Beyond these two markers, other laboratory parameters have been investigated: A hyperfibrinolytic form of disseminated intravascular coagulation (DIC), as indicated by viscoelastic testing (ROTEM) evidence of early hyperfibrinolysis, has been associated with unfavorable neurological outcomes and increased mortality after ECPR.^10^, ^11^

Neuron-specific enolase (NSE) and S100 are established biomarkers of hypoxic brain injury, but their prognostic use is primarily recommended for the 24–72 h window after circulatory arrest and their role in the earlier phase after ECPR remains uncertain. Furthermore, haemolysis, which frequently occurs during extracorporeal circulation, may lead to falsely elevated NSE levels and complicate its interpretation in patients undergoing ECPR.^4^, ^12^

Additional evidence on the prognostic role of laboratory biomarkers originates from studies in patients treated with conventional CPR rather than ECPR. Thus, serum phosphate has been associated with increased mortality and poor neurological recovery after circulatory arrest, but data specifically addressing ECPR remain limited.^13^ Cardiac biomarkers such as troponin and CK-MB have been examined as indicators of myocardial injury, yet their prognostic relevance after ECPR is inconsistent, particularly in the early post-cannulation phase.^14^

In neonates, nucleated red blood cells (NRBCs) have been investigated as biomarkers of hypoxic injury in perinatal asphyxia. Elevated NRBC counts in cord blood have been associated with the severity of hypoxic-ischemic encephalopathy and adverse clinical outcomes, suggesting that NRBCs may reflect the degree of systemic hypoxia and tissue injury during perinatal asphyxia.^15^

NRBCs are immature erythroid cells normally confined to the bone marrow and are typically absent from the peripheral blood of healthy adults. Their appearance in circulation beyond the neonatal period is therefore considered pathological. The mechanisms underlying NRBC release into the circulation appear to involve both hypoxic and inflammatory signaling. Hypoxia stimulates erythropoiesis through increased erythropoietin production, while inflammatory cytokines such as interleukin-3 and interleukin-6 further promote hematopoietic activation. These processes may accelerate the release of immature erythroid cells, including NRBCs, from the bone marrow into the peripheral blood.^16^

Clinical observations support a link between hypoxia and NRBC emergence. In critically ill patients, low arterial oxygen partial pressure has been shown to precede the appearance of NRBCs in peripheral blood and to be associated with increased mortality.^17^ Experimental data show that NRBC counts begin to rise 4 h after induced hypoxemia in animal models.^18^

We hypothesized that early NRBC elevation after ECPR reflects systemic hypoxia of sufficient severity to be associated with poor neurological outcome.

As the prognostic value of NRBCs in ECPR patients has not yet been investigated, we conducted a retrospective analysis assessing routinely available laboratory values obtained within the first six hours after admission and their associations with neurological outcome.

## Methods

The study was approved by the ethics committee of Charité—Universitätsmedizin Berlin (EA2/066/23) on June 6, 2025, and was conducted in accordance with the principles of the Declaration of Helsinki. The requirement for informed consent was waived because of the retrospective nature of the study.

### Patient selection

This retrospective single-center dual-site study was conducted at Charité–Universitätsmedizin Berlin and included patients treated at two hospital sites: Campus Virchow-Klinikum and Campus Charité Mitte. Between January 2020 and December 2024, all adult patients (≥18 years) who experienced either in-hospital (IHCA) or out-of-hospital circulatory arrest (OHCA), failed to achieve return of spontaneous circulation (ROSC) despite guideline-based advanced life support, and in whom venoarterial extracorporeal membrane oxygenation (VA-ECMO) was initiated during ongoing cardiopulmonary resuscitation were included. The decision to initiate eCPR followed predefined institutional eligibility criteria based on our center’s eCPR protocol. Patients were not considered candidates for eCPR in cases of unwitnessed cardiac arrest, absence of bystander CPR, an estimated low-flow time exceeding 60 min at the time of presentation for eCPR, or known active malignancy. Patients were enrolled irrespective of the underlying cause of circulatory arrest.

### Data collection

Resuscitation parameters, laboratory values, and clinical outcomes were obtained retrospectively from the institutional electronic medical records. Baseline demographics, details of the circulatory arrest event, and ECMO-related variables were systematically documented.

Neurological outcome was assessed using the Cerebral Performance Category (CPC) score.^19^ A poor outcome was defined as death before definitive neuroprognostic assessment or an unfavorable neurological status (CPC 3–5) at hospital discharge. CPC 1–2 at hospital discharge was defined as good outcome.

### Blood sampling and laboratory parameters analyzed

Plasma pH and lactate values, if available, were collected immediately prior to cannulation. To assess early follow-up lactate, the measurement obtained within four to six hours after admission was recorded. If multiple values were available, the value closest to six hours was used. All other routinely available laboratory parameters were included if obtained within the first six hours after admission. These parameters usually included: Blood gas analysis comprising initial pH, initial lactate, and follow-up lactate at 6 h. Clinical chemistry including liver function tests (aspartate aminotransferase (AST), alanine aminotransferase (ALT), gamma-glutamyltransferase (GGT), alkaline phosphatase (AP), and total bilirubin), renal function parameters (creatinine and urea), electrolytes and minerals (phosphate, total corrected calcium, albumin), lactate dehydrogenase (LDH) and lipase. Cardiac biomarkers included creatine kinase (CK), creatine kinase MB (absolute and relative values), troponin T, and N-terminal pro-B-type natriuretic peptide (NT-proBNP).

Inflammatory and coagulation markers comprised C-reactive protein (CRP), procalcitonin (PCT), fibrinogen, antithrombin, international normalized ratio (INR), and D-dimers.

Hematology and blood count included white blood cells (WBC), NRBCs, hemoglobin (Hb), hematocrit (Hct), and platelet count (Plt). NRBC/100 WBCs was calculated as (NRBC/WBC) × 100.

In cases of multiple measurements during this interval, the most clinically adverse (“worst”) value was selected for analysis.

### Statistical analysis

Baseline characteristics were summarized separately by outcome group (good vs. poor outcome). Continuous variables are reported as median with interquartile range (IQR), and categorical variables as counts and percentages. Missing values were documented as counts and proportions.

For comparisons of laboratory values between outcome groups, effect size Cliff’s Delta (Δ) was reported. Cliff’s Δ was calculated as a robust, non-parametric effect size quantifying the difference between the probability that a randomly selected observation from one group is larger than a randomly selected observation from the other group and the reverse probability. Cliff’s Δ ranges from −1 to +1, where values close to 0 indicate little to no difference between groups, positive values indicate higher measurements in the poor-outcome group, and negative values indicate higher measurements in the good-outcome group.

For laboratory variables showing the strongest group differences, the ability to discriminate between good and poor outcome was further evaluated using receiver operating characteristic (ROC) curves. The area under the ROC curve (AUC) was calculated as a measure of discriminative performance. An AUC of 0.5 indicates no discriminative ability, whereas values approaching 1.0 reflect stronger separation between outcome groups.

Poor outcome was defined as the positive condition in all ROC analyses. For each laboratory marker, two diagnostic operating points were determined: (i) a rule-in threshold, defined by ≥90% specificity (minimizing false-positive classifications) and (ii) a rule-out threshold, defined by ≥90% sensitivity (minimizing false-negative classifications). If multiple thresholds fulfilled a given criterion, the value with the highest complementary performance (sensitivity or specificity) was selected. Sensitivity, specificity, and corresponding confusion matrices were reported for each selected threshold.

Additional logistic regression analyses were performed to explore the association between early laboratory markers and neurological outcome. Poor neurological outcome served as the dependent variable. Two sets of analyses were conducted. First, biomarkers were analyzed as continuous variables (NRBC/100 WBC, lactate, and pH), together with selected clinical variables (shockable rhythm and time to ECMO). Second, biomarkers were analyzed using predefined thresholds derived from the ROC analyses. Odds ratios (ORs) with 95% confidence intervals (CIs) were calculated. All analyses were performed as complete-case analyses.

Pairwise correlations between continuous variables were assessed using Spearman correlation (*ρ*) coefficient and visualized with pairplots and a correlation heatmap.

All analyses were performed using R (version 4.3.3)^20^ with the packages pROC,^21^ and effsize.^22^ Graphical representations were created using ggplot2^23^ and Seaborn.^24^

## Results

Between January 2020 and December 2024, a total of 542 patients under resuscitation were presented for ECPR assessment. Among them, 36 patients achieved sustained ROSC, and 309 patients died during resuscitation without ECPR initiation.

197 consecutive patients underwent ECPR and were included in the analysis. The median age of ECPR patients was 55 years [IQR 46–61], and 85.3% were male. A shockable initial rhythm was present in 58.4%, and the median time from circulatory arrest to ECMO initiation was 75 min [60–82]. Most arrests occurred out-of-hospital (91.4% OHCA).

**Fig. 1:**
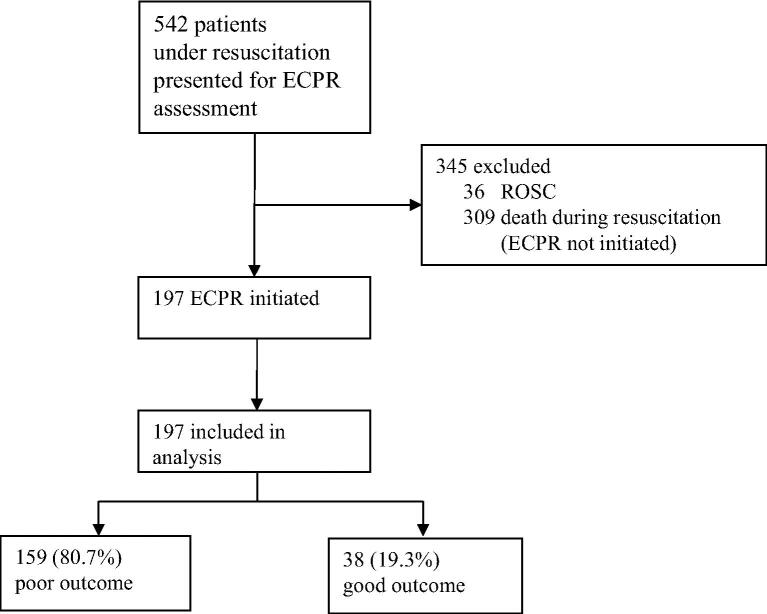
**Flowchart of the study, depicting patient screening, enrollment, and allocation within the study cohort**.

Among them, 159 (80.7%) had a poor outcome, while 38 (19.3%) achieved a good outcome (Fig. 1).

### Baseline characteristics stratified by outcome

Baseline characteristics between patients with poor and good neurological outcome were largely comparable (Table 1). Patients with poor outcome were slightly more often male and more frequently had out-of-hospital circulatory arrest. In contrast, a shockable initial rhythm was considerably more common among those with good outcome. Time to ECMO initiation and rates of witnessed arrest or bystander CPR were similar between groups.

**Table 1:** Baseline characteristics stratified by outcome.

**Characteristic**	**Poor outcome** ***N* = 159 (80.7%)**	**Good outcome** ***N* = 38 (19.3%)**	**Missing values**
Age (years)	55 [46–61]	55.5 [45.5–60]	0 (0.0%)
Male	139 (87.4%)	29 (76.3%)	0 (0.0%)
Out of hospital circulatory arrest	148 (93.1%)	32 (84.2%)	0 (0.0%)
Time to ECMO (minutes)	75 [60–83]	70 [60–79.5]	25 (12.7%)
Bystander cardiopulmonary resuscitation	125 (83.9%)	34 (89.5%)	10 (5.1%)
Witnessed arrest	129 (89.0%)	34 (91.9%)	15 (7.6%)
First rhythm shockable	83 (54.2%)	28 (75.7%)	7 (3.6%)
**First rhythm**			
Asystole	27 (17.6%)	0 (0.0%)	7 (3.6%)
Pulseless electrical activity	43 (28.1%)	9 (24.3%)	7 (3.6%)
Ventricular fibrillation/tachycardia	83 (54.2%)	28 (75.7%)	7 (3.6%)
**Cause**			
Coronary artery disease	80 (50.3%)	21 (55.3%)	0 (0.0%)
Pulmonary embolism	9 (5.7%)	6 (15.8%)	0 (0.0%)
Arrhythmogenic	8 (5.0%)	5 (13.2%)	0 (0.0%)
Other cardiac	8 (5.0%)	2 (5.3%)	0 (0.0%)
Hypothermia	2 (1.3%)	3 (7.9%)	0 (0.0%)
Other shock	3 (1.9%)	1 (2.6%)	0 (0.0%)
Hypoxia	8 (5.0%)	0 (0.0%)	0 (0.0%)
Intracranial bleeding	3 (1.9%)	0 (0.0%)	0 (0.0%)
Aortic dissection	6 (3.8%)	0 (0.0%)	0 (0.0%)
Unknown	32 (20.1%)	0 (0.0%)	0 (0.0%)

Values are medians (IQR) or *N* = number (%);

Regarding etiology, coronary causes predominated in both groups. However, pulmonary embolism, arrhythmogenic, and hypothermic origins were relatively more frequent among patients with good outcome, whereas unclear or hypoxic causes were more frequent in those with poor outcome.

### Laboratory markers associated with outcome

**Fig. 2:**
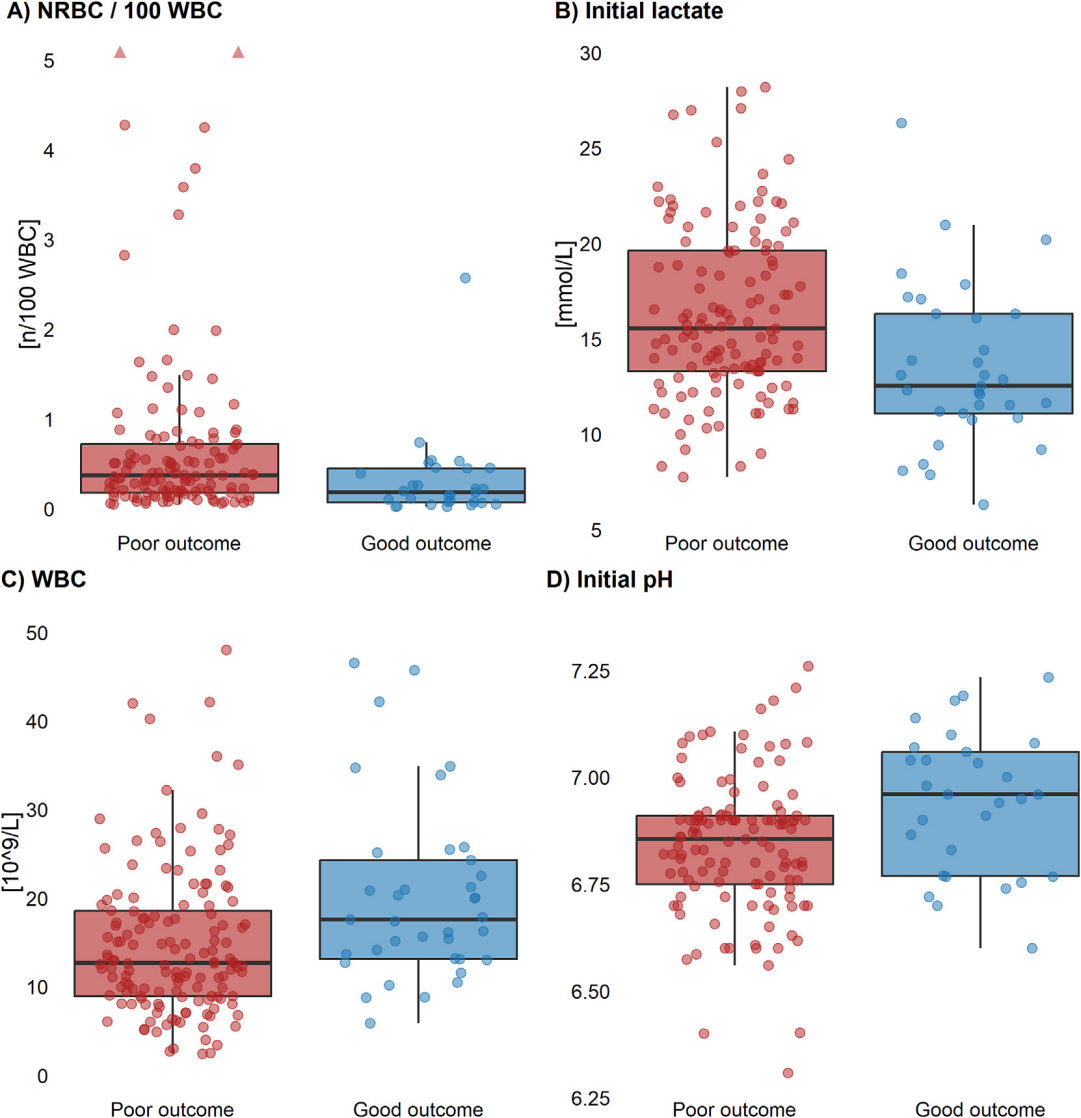
**Laboratory parameters with the largest differences between patients with poor (red) and good (blue) outcomes. Panel A shows NRBC/100 WBCs [*n*/100 WBC]. The y-axis is truncated at 5, and upward red triangles mark observations exceeding this limit. Panel B displays initial lactate [mmol/L] stratified by outcome. Panel C presents WBC [10^9^/L] stratified by outcome. Panel D shows initial pH stratified by outcome**. (For interpretation of the references to color in this figure legend, the reader is referred to the web version of this article.)

Among all laboratory parameters, the strongest associations with outcome were observed for NRBCs/100 WBCs, followed by initial lactate, WBC count, initial pH, follow-up lactate and phosphate (Fig. 2, Table 2).

**Table 2:** Prognostic associations of early routine laboratory parameters after extracorporeal cardiopulmonary resuscitation.

**Marker**	**Poor outcome**	**Good outcome**	**Effect size/confidence interval**
NRBC/100 WBCs [*n*/100 WBC]	0.37 [0.18–0.72] MV: 32 (20.1%)	0.19 [0.07–0.45] MV: 9 (23.7%)	Cliff’s Δ: 0.39 [0.15, 0.58]
Initial lactate [mmol/L]	15.5 [13.3–19.7] MV: 32 (20.1%)	12.5 [11.1–16.3] MV: 5 (13.2%)	Cliff’s Δ: 0.38 [0.16, 0.57]
WBC [10^9^/L]	12.68 [8.95–18.57] MV: 12 (7.5%)	17.59 [13.16–24.28] MV: 1 (2.6%)	Cliff’s Δ: −0.36 [−0.53, −0.17]
Initial pH	6.86 [6.75–6.91] MV: 38 (23.9%)	6.96 [6.77–7.06] MV: 9 (23.7%)	Cliff’s Δ: −0.34 [−0.55, −0.09]
Follow-up lactate [mmol/L]	11.7 [7.9–15.0] MV: 47 (29.6%)	7.6 [5.6–12.1] MV: 5 (13.2%)	Cliff’s Δ: 0.33 [0.10, 0.52]
Phosphate [mmol/L]	3.33 [2.79–4.02] MV: 27 (17.0%)	3.09 [1.94–3.54] MV: 3 (7.9%)	Cliff’s Δ: 0.29 [0.07, 0.47]

Values are median (IQR) or *N* = number (%), MV: Missing value, Cliff’s Δ: Cliff’s delta effect size.

NRBC/100 WBCs showed the largest effect size: poor outcome had higher values (0.37/100 WBCs [0.18–0.72] vs. 0.19/100 WBCs [0.07–0.45]; Cliff’s Δ 0.39, 95% CI 0.15–0.58), reflecting a systematic shift towards elevated NRBC levels in this group. Considering the absolute number of NRBCs in counts per 10^9^/L, values within the first six hours after admission were also higher in patients with poor outcome (0.04 × 10^9^/L [0.02–0.07]) compared to those with good outcome (0.02 × 10^9^/L [0.01–0.04]; Cliff’s Δ = 0.29 [0.06–0.50]) (Supplemental Fig. 1A).

Similarly, initial lactate was markedly increased in patients with poor outcome (15.5 mmol/L [13.3–19.7] vs. 12.5 mmol/L [11.1–16.3]; Cliff’s Δ 0.38, 95% CI 0.16–0.57).

In contrast, WBC counts were higher in patients with good outcome (17.6 × 10^9^/L [13.2–24.3] vs. 12.7 × 10^9^/L [9.0–18.6]; Cliff’s Δ −0.36, 95% CI −0.53 to −0.17).

Initial pH was modestly higher in the good outcome group (6.96 [6.77–7.06] vs. 6.86 [6.75–6.91]; Cliff’s Δ −0.34, 95% CI −0.55 to −0.09), consistent with less severe acidosis in this group.

Follow-up lactate at 6 h further accentuated these differences: while levels declined in good outcomes (7.6 mmol/L [5.6–12.1]), they remained elevated in poor outcome (11.7 mmol/L [7.9–15.0]; Cliff’s Δ 0.33, 95% CI 0.10–0.52), highlighting impaired clearance among poor outcome patients.

Phosphate concentrations were modestly but consistently higher in those with poor outcome (3.33 mmol/L [2.79–4.02] vs. 3.09 mmol/L [1.94–3.54]; Cliff’s Δ 0.29, 95% CI 0.07–0.47).

All other laboratory parameters are reported in the Supplemental Material (Supplemental Table 1). Differences between outcome groups were small, with effect sizes (Cliff’s Δ) not exceeding 0.22 in absolute value.

### Discriminative performance of laboratory parameters

ROC analyses confirmed moderate overall discrimination across the most outcome-associated parameters, with AUC values ranging from 0.64 to 0.70 (Table 3).

**Table 3:** Predictive performance of laboratory markers at rule-in thresholds (≥90% specificity).

**Marker**	**AUC** **(95% CI)**	**Threshold**	**Sensitivity**	**Specificity**	**True/false poor outcome**	**PPV**^a^	***N***
NRBC/100 WBCs [*n*/100 WBCs]	0.70 (0.59–0.80)	0.54	0.33	0.93	42/2	0.95	156
Initial lactate [mmol/L]	0.69 (0.59–0.80)	18.43	0.31	0.909	39/3	0.93	160
WBC [10^9^/L]	0.68 (0.59–0.80)	10.16	0.33	0.919	48/3	0.94	184
Initial pH	0.67 (0.55–0.79)	6.72	0.20	0.931	24/2	0.92	150
Follow-up lactate [mmol/L]	0.66 (0.56–0.77)	14.7	0.27	0.909	30/3	0.91	145
Phosphate [mmol/L]	0.64 (0.54–0.75)	3.78	0.35	0.914	46/3	0.94	167

a Positive predictive value.

Among all investigated laboratory markers, NRBC/100 WBCs demonstrated the highest discriminative performance and the highest positive predictive value (PPV) for the rule-in threshold (≥90% specificity).

**Fig. 3:**
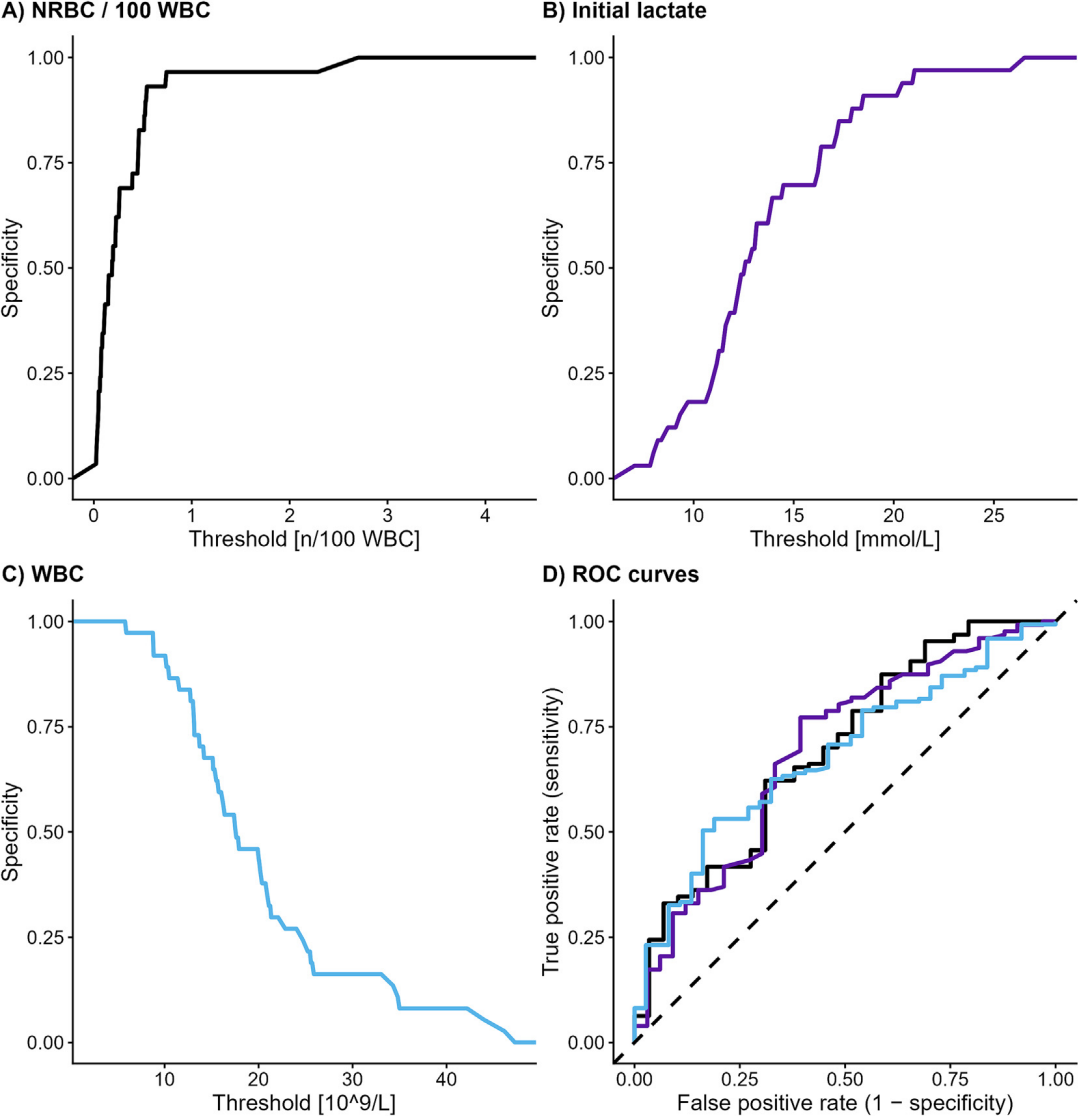
**Receiver operating characteristic (ROC) and specificity analyses, with cut-offs calculated to achieve ≥90% specificity. Panel A shows the specificity curve for NRBC/100 WBCs, panel B the specificity curve for initial lactate, and panel C the specificity curve for WBC. Panel D presents the combined ROC curves of these three markers**.

The rule-in threshold for NRBC/100 WBCs (≥0.54 *n*/100 WBC; AUC 0.70 [95% CI 0.59–0.80]; specificity 93%, sensitivity 33%) identified 42 true-positive (positive = poor outcome) and 2 false-positive cases, corresponding to a PPV of 0.95. The corresponding specificity curve is shown in Fig. 3, panel A.

In a sensitivity analysis restricted to OHCA patients, the diagnostic performance of NRBC/100 WBCs remained similar. The rule-in threshold showed a sensitivity of 30.8% and a specificity of 96.0%, with a positive predictive value of 0.97 (37 true poor outcomes and 1 false poor outcome, *n* = 145).

When stratified by quartiles of NRBC/100 WBC measured within the first six hours after eCPR, the proportion of poor outcome increased stepwise from 69.2% in the lowest quartile to 94.9% in the highest quartile, indicating a graded association across the biomarker distribution (Supplemental Table 2).

In contrast, absolute NRBC counts showed weaker discriminative performance (AUC 0.65 [95% CI 0.53–0.76]). At the rule-in threshold of 0.085 × 10^9^/L, 24 true-positive and 2 false-positive cases were identified (PPV 0.92). The corresponding specificity curve is shown in Supplemental Fig. 1B.

Initial lactate (≥18.4 mmol/L; AUC 0.69 [0.59–0.80]; specificity 91%, sensitivity 31%) yielded 39 true-positive and 3 false-positive cases (PPV 0.93; Fig. 3, panel B). WBC count demonstrated comparable discrimination (AUC 0.68 [95% CI 0.59–0.80]); the rule-in threshold (≤10.2 × 10^9^/L; specificity 92%, sensitivity 33%) correctly identified 48 true-positive and 3 false-positive cases (PPV 0.94; Fig. 3, panel C).

Initial pH (AUC 0.67 [95% CI 0.55–0.79]), follow-up lactate at six hours (AUC 0.66 [0.56–0.77]), and phosphate (AUC 0.64 [0.54–0.75]) showed similar rule-in performance, achieving specificities of 91–93% and PPVs between 0.91 and 0.94 (Table 3).

High-sensitivity rule-out thresholds (≥90% sensitivity) were additionally derived for all markers but showed limited discriminative accuracy and frequent misclassification of good-outcome patients, with only 6–11 true-good and 11–14 false-good cases. Detailed results are provided in Supplemental Table 3 (predictive performance of laboratory markers at rule-out thresholds ≥90% sensitivity), Supplemental Fig. 2 (confusion matrices at rule-in thresholds ≥90% specificity), and Supplemental Fig. 3 confusion matrices at rule-out thresholds ≥90% sensitivity).

### Univariable logistic regression analysis

Univariable logistic regression analyses were performed to evaluate the association between early laboratory markers and poor neurological outcome.

When analyzed as continuous variables, higher lactate levels were associated with increased odds of poor neurological outcome (OR 1.18 per mmol/L increase, 95% CI 1.06–1.32; *p* = 0.002), whereas higher pH values were associated with lower odds of poor outcome (OR 0.02 per unit increase, 95% CI 0.001–0.28; *p* = 0.004). NRBC/100 WBC showed a similar direction of association with poor outcome but with wider confidence intervals (OR 3.41 per unit increase, 95% CI 0.93–12.46; *p* = 0.064). Time to ECMO showed little evidence of an association with outcome (OR 1.01 per minute increase, 95% CI 0.99–1.03; *p* = 0.309). These analyses are presented in Supplemental Table 4.

When biomarkers were evaluated using predefined thresholds derived from the ROC analyses, NRBC ≥0.54 was associated with higher odds of poor neurological outcome (OR 6.67, 95% CI 1.51–29.39; *p* = 0.012), and lactate ≥18.43 mmol/L showed a similar pattern (OR 4.43, 95% CI 1.28–15.40; *p* = 0.019). In contrast, the pH threshold ≤6.72 showed weaker evidence of an association with outcome (OR 2.49, 95% CI 0.70–8.86; *p* = 0.159). A shockable rhythm was associated with lower odds of poor neurological outcome (OR 0.38, 95% CI 0.17–0.86; *p* = 0.020). These results are summarized in Supplemental Table 5.

### Overlap and correlations between laboratory markers

Among NRBC/100 WBCs–positive patients, overlap with other unfavorable markers defined by rule-in thresholds (Table 3) was limited: 38.6% had elevated initial lactate, 56.8% low WBCs, 20.5% low initial pH, 31.8% positive follow-up lactate, and 27.3% elevated phosphate (Supplemental Table 6). NRBC/100 WBCs showed a moderate positive correlation with initial lactate (*ρ* = 0.42), a moderate negative correlation with WBCs (*ρ* = −0.41), and a weak negative correlation with pH (*ρ* = −0.24).

Only 26.9% of patients with positive absolute NRBC counts met the WBC rule-in criterion, and the correlation between absolute NRBC counts and WBC was negligible (*ρ* = −0.02).

WBC correlated only weakly with other parameters, such as initial lactate (*ρ* = −0.07) and pH (*ρ* = 0.02). Pairplots are shown in Supplemental Fig. 4 and the Spearman correlation heatmap is shown in Supplemental figure 5.

Using the predefined ROC-derived thresholds, combined rule-in definitions achieved maximal specificity for poor outcome. The combination of NRBC ≥0.54 per 100 WBCs with initial lactate ≥18.43 mmol/L identified 17 patients with poor outcome without false-positive classifications (PPV 1.0), while NRBC ≥0.54 with initial pH ≤6.72 identified 9 patients with poor outcome without false positives (PPV 1.0) (Supplemental Table 7).

Patients fulfilling the NRBC rule-in criterion despite lactate or pH values below their respective thresholds still showed a high rate of poor outcome (14/15 [93.3%] and 21/22 [95.5%], respectively), whereas combined rule-out definitions showed high sensitivity (96.1% and 98.0%) but low specificity and therefore did not reliably predict favorable outcome (Supplemental Table 8).

## Discussion

This retrospective single-center, dual-site study examined routinely available laboratory parameters within the first six hours after ECPR for refractory circulatory arrest and, to our knowledge, is the first to evaluate NRBCs in this context. NRBCs, lactate, WBCs, pH, follow-up lactate and phosphate were associated with poor neurological outcome. Although the overall discriminative power of individual markers was moderate (AUC range, 0.64–0.70), high positive predictive values at stringent rule-in thresholds allowed identification of patients with poor outcome, whereas rule-out performance remained limited.

### Nucleated red blood cells as early prognostic markers after ECPR

Among all investigated parameters, NRBC/100 WBCs was most strongly associated with neurological outcome, extending previous ICU observations that link NRBC elevation to mortality.^25^, ^26^, ^27^, ^28^ Comparable findings have been reported in neonates, where NRBC/100 WBCs are used as a simple early indicator of perinatal asphyxia severity and prognosis.^29^ In that context, measurements obtained within the first six hours after birth strongly correlated with the development of hypoxic–ischemic encephalopathy and subsequent brain injury.^30^ This parallels our findings, suggesting that NRBC elevation may represent a general marker of severe hypoxic stress and is associated to early neuronal injury across different patient populations.

In our cohort, this association likely reflects a more pronounced or prolonged hypoxic burden before ECPR, triggering bone marrow stimulation and NRBC release.^17^ Normally absent in adults, NRBCs appear when hypoxia stabilizes HIF-2α and drives erythropoietin-mediated expansion of erythroid progenitors.^31^ This pattern suggests that NRBC elevation reflects a pathophysiological dimension distinct from metabolic derangement. Although NRBC levels correlated moderately with lactate, the overlap with other unfavorable metabolic markers was limited, indicating that NRBC positivity identifies a subgroup with poor outcome not primarily driven by metabolic factors.

Consistent with this pattern, NRBCs showed only limited evidence of an association with outcome when analyzed as a continuous variable in logistic regression. In contrast, applying the predefined rule-in threshold revealed substantially higher odds of poor neurological outcome (OR 6.67) compared with the corresponding estimates for lactate and pH. This pattern suggests that NRBC elevation may primarily identify a small subgroup of patients with particularly high risk rather than reflecting a gradual increase in risk across the full range of values.

### Interrelation of NRBC and WBC responses after ECPR

NRBCs have been reported inconsistently across studies, either as per 100 WBCs, absolute concentrations, or in both formats. In our cohort, both measures were associated with poor neurological outcome, but NRBC/100 WBCs showed superior discrimination. Because this measure inherently combines erythroid and leukocytic components, understanding the behavior of WBCs themselves is essential for interpretation.

Lower WBC counts within the first six hours were linked to poor outcome and interestingly did not correlate with any other marker. This observation aligns with prior reports associating lymphopenia with poor recovery after conventional CPR,^32^ suggesting that an early leukocytic response may indicate preserved immune competence. Leukocyte kinetics after ECPR, however, has not been systematically characterized to date, and the lack of differential counts limited further interpretation.

The superior performance of NRBC/100 WBCs likely reflects the imbalance between erythroid and myeloid activation. Leukocyte activation after reperfusion represents a rapid inflammatory response,^33^, ^34^ whereas NRBC release reflects erythropoietin-driven marrow stimulation in response to hypoxia.^31^ These processes capture largely independent biological axes.

Although the ratio could theoretically be influenced by variations in WBC, NRBC-counts and WBC were not correlated, and the overlap between NRBC-counts-positive and WBC-positive patients was minimal, suggesting that the prognostic contribution of NRBC/100 WBCs is not simply driven by leukocyte-dependent effects. Normalization to WBC may therefore attenuate inflammatory confounding and help isolate the signal of hypoxia-mediated marrow activation, although this interpretation remains cautious given the observational nature of our data.

When combined with metabolic thresholds, NRBC positivity identified a subgroup with extremely high risk of poor outcome, with the combined rule-in definitions achieving a PPV of 100% in our cohort. At the same time, the presence of patients fulfilling the nRBC rule-in criterion despite lactate or pH values below the respective thresholds suggests that nRBCs may capture injury pathways not fully reflected by metabolic markers alone, thereby augmenting traditional biomarkers in early risk stratification after eCPR.

Together, these findings suggest that disproportionate erythroid activation relative to leukocytic mobilization, reflected by NRBC/100 WBCs, marks severe hypoxia and poor prognosis, whereas a concurrent leukocytic response may indicate a more balanced and potentially compensatory reaction.

### Confirmatory analysis of established metabolic prognostic markers

Many clinical chemistry parameters reflecting organ dysfunction, such as markers of cardiac, renal or hepatic injury, typically rise only hours to days after ischemia–reperfusion injury. Therefore, the limited associations observed for several baseline parameters in our study likely reflect the early six-hour sampling window rather than a lack of prognostic relevance.

Elevated initial lactate levels were associated with poor neurological outcome after ECPR, consistent with previous studies. Reported thresholds associated with poor outcome vary, with cut-offs of 14 mmol/L^5^ and 15.1 mmol/L,^6^ while the German consensus paper defines concentrations >20 mmol/L as a negative but not absolute criterion for ECPR initiation.^27^ We tested these published thresholds within our cohort and observed a progressive decline in favorable outcomes with rising lactate levels (Supplemental Table 9): at ≥14 mmol/L, 80 patients were correctly and 11 (12.1%) falsely classified as poor outcome; at ≥15.1 mmol/L, 68 and 10 (12.8%); and at ≥20 mmol/L, 27 and 3 (10.0%), respectively. A threshold of 18.4 mmol/L yielded 91% specificity (39 true, 3 false poor predictions). Overall, lactate showed only moderate discriminatory value, and rigid thresholds may lead to inappropriate exclusion from ECPR.

Initial pH was only modestly associated with neurological outcome, with considerable overlap between groups. Evidence for pH as a selection criterion remains limited; pH was identified as a potential prognostic factor but noted substantial heterogeneity and no consistent threshold across studies.^9^ A pH ≤6.8, cited in the German ECPR consensus statement,^35^ was not predictive in our cohort, as 8 of 56 patients (14.3%) with pH ≤6.8 achieved good outcome. The prognostic value of pH likely improves when combined with other variables such as the initial cardiac rhythm: pH >7 together with a shockable rhythm independently predicted favorable outcomes.^8^

Higher serum phosphate concentrations were modestly but consistently associated with poor neurological outcome after ECPR. A threshold of 3.78 mmol/L correctly identified 46 patients with poor outcome and misclassified 3. Elevated phosphate after circulatory arrest has previously been linked to adverse outcomes, likely reflecting cellular injury and release of intracellular phosphate during ischemia–reperfusion, serving as a marker of tissue.^36^

### Strengths and limitations

This dual-center ECPR cohort from two high-volume institutions provides a well-characterized sample (*n* = 197) with standardized neurological outcome assessment. We systematically analyzed a comprehensive panel of early laboratory parameters using robust nonparametric effect sizes and clinically interpretable ROC-derived thresholds. NRBCs were evaluated both as absolute concentrations and normalized per 100 WBC, allowing assessment of lineage-specific marrow responses. Confirmatory results for established markers such as pH, lactate, and phosphate support the internal validity and overall data quality of the dataset. A validation of previously published lactate and pH cut-offs was performed within our cohort, enabling direct comparison with established prognostic thresholds. Missing data and classification performance were transparently reported.

Limitations include the retrospective design with potential residual confounding, variable sampling times, and incomplete datasets. Because the cohort included both out-of-hospital and in-hospital cardiac arrest patients, underlying conditions or ongoing treatments before arrest may have influenced NRBC levels.

Mechanistic biomarkers and differential leukocyte counts were unavailable, precluding detailed characterization of marrow and immune activation. All thresholds were derived in-sample without external validation, and the single healthcare-system setting may limit generalizability. Subgroup analyses were further constrained by small sample size.

## Conclusion

In this retrospective dual-site ECPR cohort, several routinely available laboratory parameters obtained within the first six hours after admission were associated with neurological outcome. Among all investigated markers, NRBC/100 WBCs showed the highest discriminative performance and the best positive predictive value for poor outcome. All analyzed showed weak performance at predicting patients with good outcome.

NRBCs were elevated and WBC counts reduced in patients with poor neurological outcome, indicating distinct but complementary hematologic responses to hypoxemia and post-resuscitation injury.

Lactate prior to ECMO cannulation provided stronger discriminative performance than pH but, like all tested parameters, should be interpreted within the overall clinical context and not as an absolute criterion again ECPR initiation.

## CRediT authorship contribution statement

**Julius Valentin Kunz:** Writing – review & editing, Writing – original draft, Visualization, Validation, Supervision, Resources, Project administration, Methodology, Investigation, Formal analysis, Data curation, Conceptualization. **Mareen Pigorsch:** Writing – review & editing, Methodology, Investigation, Formal analysis. **Jens Nee:** Writing – review & editing, Resources, Conceptualization. **Lilly Koppelkamm:** Writing – review & editing, Resources. **Teresa Carola Juchem:** Writing – review & editing, Resources. **Roland Körner:** Writing – review & editing, Resources. **Kai-Uwe Eckardt:** Writing – review & editing, Resources. **Philipp Enghard:** Writing – review & editing, Resources. **Jan Matthias Kruse:** Writing – review & editing, Supervision, Resources, Methodology, Investigation, Formal analysis, Conceptualization. **Abakar Magomedov:** Writing – review & editing, Supervision, Resources, Methodology, Formal analysis, Conceptualization.

## Ethics approval and consent to participate

The study received approval from the ethics committee of Charité—Universitätsmedizin Berlin (EA2/066/23) on June 6, 2025. It was carried out in accordance with the Declaration of Helsinki. Owing to its retrospective design, the requirement for informed consent was waived.

## Funding

This research did not receive any specific grant from funding agencies in the public, commercial, or not-for-profit sectors.

## Declaration of competing interest

The authors declare no competing interests.

## Data Availability

Due to restrictions imposed by the German Federal Data Protection Act (Bundesdatenschutzgesetz, BDSG), the underlying patient data cannot be made publicly available. Deidentified individual data may be shared upon reasonable request for scientifically sound purposes and in compliance with applicable data protection regulations. Data access requests can be directed to Julius-Valentin.Kunz@charite.de and will require a data access agreement authorized by Charité–Universitätsmedizin Berlin.
