# Supplementary material for: Nucleated red blood cells as early predictors of poor neurological outcome after extracorporeal cardiopulmonary resuscitation: a retrospective dual-site cohort study of early laboratory markers
Source: Resusc Plus. 2026 Apr 24;29:101340. doi: 10.1016/j.resplu.2026.101340 (PMC13156717; doi:10.1016/j.resplu.2026.101340)
Supplement: Supplementary Data 1 [file mmc1.pdf]

Supplemental Material

Nucleated Red Blood Cells as Early Predictors of Poor Neurological Outcome After Extracorporeal Cardiopulmonary Resuscitation: A Retrospective Dual-Site Cohort Study of Early Laboratory Markers

Address for correspondence:

Julius Valentin Kunz

Department of Nephrology and Medical Intensive Care

Charité – Universitätsmedizin Berlin,

Augustenburger Platz 1

13353 Berlin, Germany

Email: Julius-Valentin.Kunz@charite.de

Table of content

1. **Supplemental Table 1.** Prognostic associations of further early routine laboratory parameters after extracorporeal cardiopulmonary resuscitation p. 3
2. **Supplemental Figure 1.** Absolute NRBC counts and specificity) p. 5
3. **Supplemental Table 2**. Neurological outcome across quartiles of NRBC/100 WBC measured within the first six hours after eCPR p. 6
4. **Supplemental Table 3.** Predictive performance of laboratory markers at rule-out thresholds (≥ 90 % sensitivity) p. 6
5. **Supplemental Figure 2.** Confusion matrices at rule-in thresholds (≥ 90 % specificity) p. 7
6. **Supplemental Figure 3.** Confusion matrices at rule-out thresholds (≥ 90 % sensitivity) p. 8
7. **Supplemental Table 4**. Univariable logistic regression models using continuous biomarker values for prediction of poor neurological outcome p.9
8. **Supplemental Table 5**. Univariable logistic regression models using predefined biomarker thresholds for prediction of poor neurological outcome p.9
9. **Supplemental Figure 4.** Pairplot of Clinical Variables by Outcome p. 10
10. **Supplemental Figure 5.** Spearman correlation heatmap (all patients) p. 11
11. **Supplemental Table 6.** Overlap of NRBC /100 WBC positivity with other laboratory predictors by study-specific cutoffs p. 12
12. **Supplemental Table 7.** Combined biomarker rule-in and rule-out performance p. 12
13. **Supplemental Table 8.** Patients fulfilling the nRBC rule-in criterion without metabolic marker positivity p.13
14. **Supplemental Table 9.** Frequency of established lactate and pH thresholds among patients with good neurological outcome after ECPR p.13

Supplemental Table 1: Prognostic associations of further early routine laboratory parameters after extracorporeal cardiopulmonary resuscitation

Variable	Poor outcome<br>N=159 (80.7%)	Good outcome<br>N=38 (19.3%)	Effect size / Confidence interval
NRBC / 100 WBCs [n/100 WBC]	0.37 [0.18–0.72]<br>MV: 32 (20.1%)	0.19 [0.07–0.45]<br>MV: 9 (23.7%)	Cliff's Δ: 0.39 [0.15, 0.58]
Initial lactate [mmol/L]	15,5 [13,3–19,7]<br>MV: 32 (20.1%)	12,5 [11,1–16,3]<br>MV: 5 (13.2%)	Cliff's Δ: 0.38 [0.16, 0.57]
WBC [10^9/L]	12.68 [8.95–18.57]<br>MV: 12 (7.5%)	17.59 [13.16–24.28]<br>MV: 1 (2.6%)	Cliff's Δ: -0.36 [-0.53, -0.17]
Initial pH	6.86 [6.75–6.91]<br>MV: 38 (23.9%)	6.96 [6.77–7.06]<br>MV: 9 (23.7%)	Cliff's Δ: -0.34 [-0.55, -0.09]
Lactate kinetics [mmol/L]	11,7 [7,9–15,0]<br>MV: 47 (29.6%)	7,6 [5,6–12,1]<br>MV: 5 (13.2%)	Cliff's Δ: 0.33 [0.10, 0.52]
Phosphate [mmol/L]	3.33 [2.79–4.02]<br>MV: 27 (17.0%)	3.09 [1.94–3.54]<br>MV: 3 (7.9%)	Cliff's Δ: 0.29 [0.07, 0.47]
NRBCs [× 10^9/L]	0.04 [0.02–0.07]<br>MV: 32(20.1%)	0.02 [0.01–0.04]<br>MV: 9 (23.7%)	Cliff's Δ: 0.29 [0.06–0.50]
Urea [mg/dL]	34 [27–41]<br>MV: 15 (9.4%)	38 [31–47]<br>MV: 3 (7.9%)	Cliff's Δ: -0.218 [-0.406, -0.012]
Fibrinogen [g/L]	1.42 [0.91–2.02]<br>MV: 29 (18.2%)	1.70 [1.35–2.08]<br>MV: 1 (2.6%)	Cliff's Δ: -0.194 [-0.370, -0.004]
Platelets [10^9/L]	147 [105–198]<br>MV: 13 (8.2%)	192.00 [131–227]<br>MV: 1 (2.6%)	Cliff's Δ: -0.187 [-0.378, 0.020]
Procalcitonin [ng/mL]	0.05 [0.03–0.14]<br>MV: 24 (15.1%)	0.09 [0.04–0.54]<br>MV: 1 (2.6%)	Cliff's Δ: -0.184 [-0.384, 0.034]
NT-proBNP [pg/mL]	238 [65–852]<br>MV: 25 (15.7%)	375.00 [140–1168]<br>MV: 3 (7.9%)	Cliff's Δ: -0.183 [-0.371, 0.019]

CRP [mg/L]	1.70 [0.70–6.05]<br>MV: 13 (8.2%)	3.90 [1.25–10.52]<br>MV: 2 (5.3%)	Cliff’s Δ: -0.175 [-0.363, 0.027]
INR [-]	1.44 [1.29–2.17]<br>MV: 20 (12.6%)	1.36 [1.26–1.52]<br>MV: 2 (5.3%)	Cliff’s Δ: 0.166 [-0.030, 0.350]
CK [U/L]	734.50 [325–2583]<br>MV: 13 (8.2%)	502.00 [282–2413]<br>MV: 1 (2.6%)	Cliff’s Δ: 0.131 [-0.083, 0.335]
CK-MB [U/L]	177.00 [116–385]<br>MV: 18 (11.3%)	143.25 [99–406]<br>MV: 2 (5.3%)	Cliff’s Δ: 0.125 [-0.088, 0.328]
Lipase [U/L]	58.50 [40.25–83.00]<br>MV: 17 (10.7%)	53.00 [37.75–71.75]<br>MV: 2 (5.3%)	Cliff’s Δ: 0.12 [-0.092, 0.322]
Total bilirubin [mg/dL]	0.27 [0.17–0.43]<br>MV: 12 (7.5%)	0.38 [0.17–0.49]<br>MV: 1 (2.6%)	Cliff’s Δ: -0.114 [-0.326, 0.108]
Creatinine [mg/dL]	1.43 [1.26–1.62]<br>MV: 12 (7.5%)	1.41 [1.23–1.56]<br>MV: 1 (2.6%)	Cliff’s Δ: 0.076 [-0.126, 0.273]
Calcium [mmol/L]	2.08 [1.97–2.17]<br>MV: 30 (18.9%)	2.06 [1.96–2.14]<br>MV: 3 (7.9%)	Cliff’s Δ: 0.075 [-0.131, 0.274]
CK-MB [%]	29.00 [20.00–42.00]<br>MV: 30 (18.9%)	30.00 [14.25–45.50]<br>MV: 4 (10.5%)	Cliff’s Δ: 0.072 [-0.174, 0.310]
Calcium (corrected) [mmol/L]	2.37 [2.27–2.55]<br>MV: 33 (20.8%)	2.38 [2.28–2.47]<br>MV: 4 (10.5%)	Cliff’s Δ: 0.063 [-0.136, 0.258]
Alkaline phosphatase [U/L]	79.00 [61.00–97.75]<br>MV: 21 (13.2%)	79.00 [60.00–110.50]<br>MV: 3 (7.9%)	Cliff’s Δ: -0.061 [-0.282, 0.166]
Troponin T [ng/L]	369.00 [129–2020]<br>MV: 20 (12.6%)	219.50 [149–4445]<br>MV: 2 (5.3%)	Cliff’s Δ: 0.042 [-0.183, 0.263]
LDH [U/L]	652 [463–1238]<br>MV: 22 (13.8%)	618.00 [439–1048]<br>MV: 3 (7.9%)	Cliff’s Δ: 0.041 [-0.175, 0.254]
ALT [U/L]	169 [117–296]<br>MV: 12 (7.5%)	161 [89–455]<br>MV: 1 (2.6%)	Cliff’s Δ: 0.04 [-0.186, 0.261]
Albumin [g/L]	27.20 [21.82–30.35]<br>MV: 25 (15.7%)	25.50 [22.40–30.00]<br>MV: 1 (2.6%)	Cliff’s Δ: 0.032 [-0.177, 0.239]

Hemoglobin [g/dL]	10.90 [9.20–12.50]<br>MV: 12 (7.5%)	10.70 [8.90–12.90]<br>MV: 1 (2.6%)	Cliff's Δ: -0.029 [-0.245, 0.189]
AST [U/L]	293 [167–755]<br>MV: 16 (10.1%)	322 [155–637]<br>MV: 2 (5.3%)	Cliff's Δ: 0.02 [-0.195, 0.233]
GGT [U/L]	53.00 [33.00–100.00]<br>MV: 12 (7.5%)	54.00 [26.00–116.00]<br>MV: 1 (2.6%)	Cliff's Δ: 0.02 [-0.200, 0.238]
Antithrombin [%]	56.00 [41.00–65.25]<br>MV: 35 (22.0%)	54.00 [49.00–63.00]<br>MV: 5 (13.2%)	Cliff's Δ: -0.017 [-0.219, 0.188]
D-dimers [µg/mL]	35.00 [23.84–35.00]<br>MV: 40 (25.2%)	35.00 [21.14–35.00]<br>MV: 7 (18.4%)	Cliff's Δ: 0.007 [-0.202, 0.216]
Hematocrit [L/L]	0.34 [0.29–0.39]<br>MV: 12 (7.5%)	0.34 [0.27–0.38]<br>MV: 1 (2.6%)	Cliff's Δ: 0.001 [-0.212, 0.214]

Values are median (IQR) or N=number (%), MV: Missing value, Cliff's Δ: Cliff's delta effect size

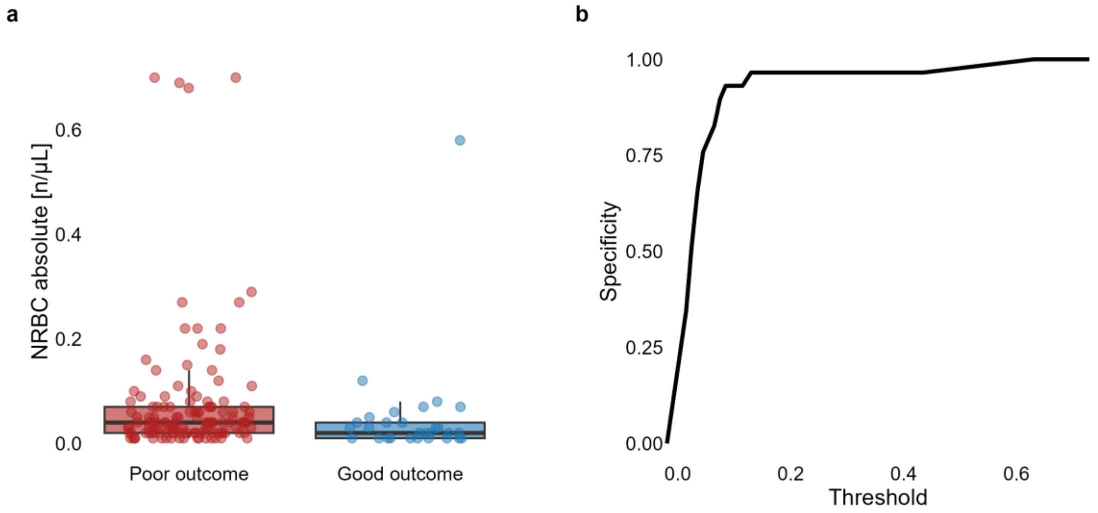

Supplemental Figure 1. Absolute nucleated red blood cell (NRBC) counts and specificity curve. Panel a displays the distribution of absolute NRBC concentrations within the first six hours after admission, shown as individual data points and overlaid boxplots stratified by neurological outcome. Panel b shows the corresponding specificity curve for poor neurological outcome across the range of NRBC thresholds.

Supplemental Table 2. Neurological outcome across quartiles of NRBC/100 WBC measured within the first six hours after eCPR

NRBC/100 WBC quartile	Range	N	Poor neurological outcome n (%)	Good neurological outcome n (%)
Q1	0.02–0.15	39	27 (69.2%)	12 (30.8%)
Q2	0.15–0.33	39	31 (79.5%)	8 (20.5%)
Q3	0.34–0.61	39	32 (82.1%)	7 (17.9%)
Q4	0.64–12.37	39	37 (94.9%)	2 (5.1%)

Supplemental Table 3: Predictive performance of laboratory markers at rule-out thresholds (≥90% sensitivity)

Marker	Threshold	Sensitivity	Specificity	True / false good outcome	n
NRBC / 100 WBC [n/100 WBC]	0.09	0.91	0.35	10 / 12 (54.5%)	156
Initial lactate [mmol/L]	11.1	0.91	0.27	9 / 12 (57.1%)	160
WBC [10^9/L]	26.5	0.91	0.16	6 / 14 (70.0%)	184
Initial pH	7.07	0.90	0.24	7 / 12 (63.2%)	150
Lactate kinetics [mmol/L]	44.0	0.90	0.24	8 / 11 (57.9%)	145
Phosphate [mmol/L]	2.03	0.91	0.31	11 / 12 (52.2%)	167

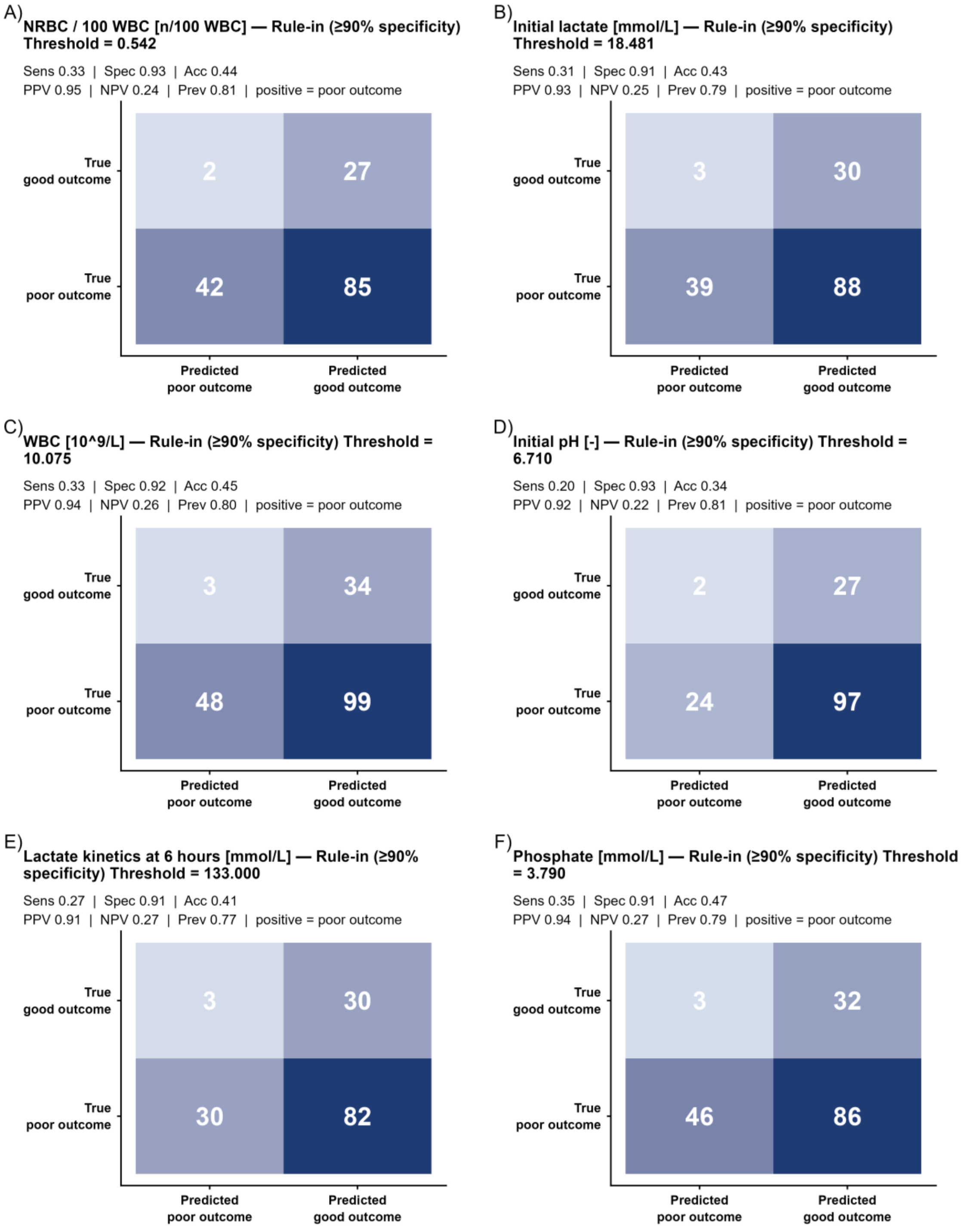

Supplemental Figure 2. Confusion matrices at rule-in thresholds (≥ 90 % specificity). *Each panel displays the confusion matrix for one laboratory marker at the rule-in threshold, defined by ≥ 90 % specificity. Poor outcome was defined as the positive condition. TP = true positives, FP = false positives, FN = false negatives, TN = true negatives, Sens = sensitivity, Spec = specificity, PPV = positive predictive value, NPV = negative predictive value, Acc = overall accuracy, Prev = prevalence of poor outcome.*

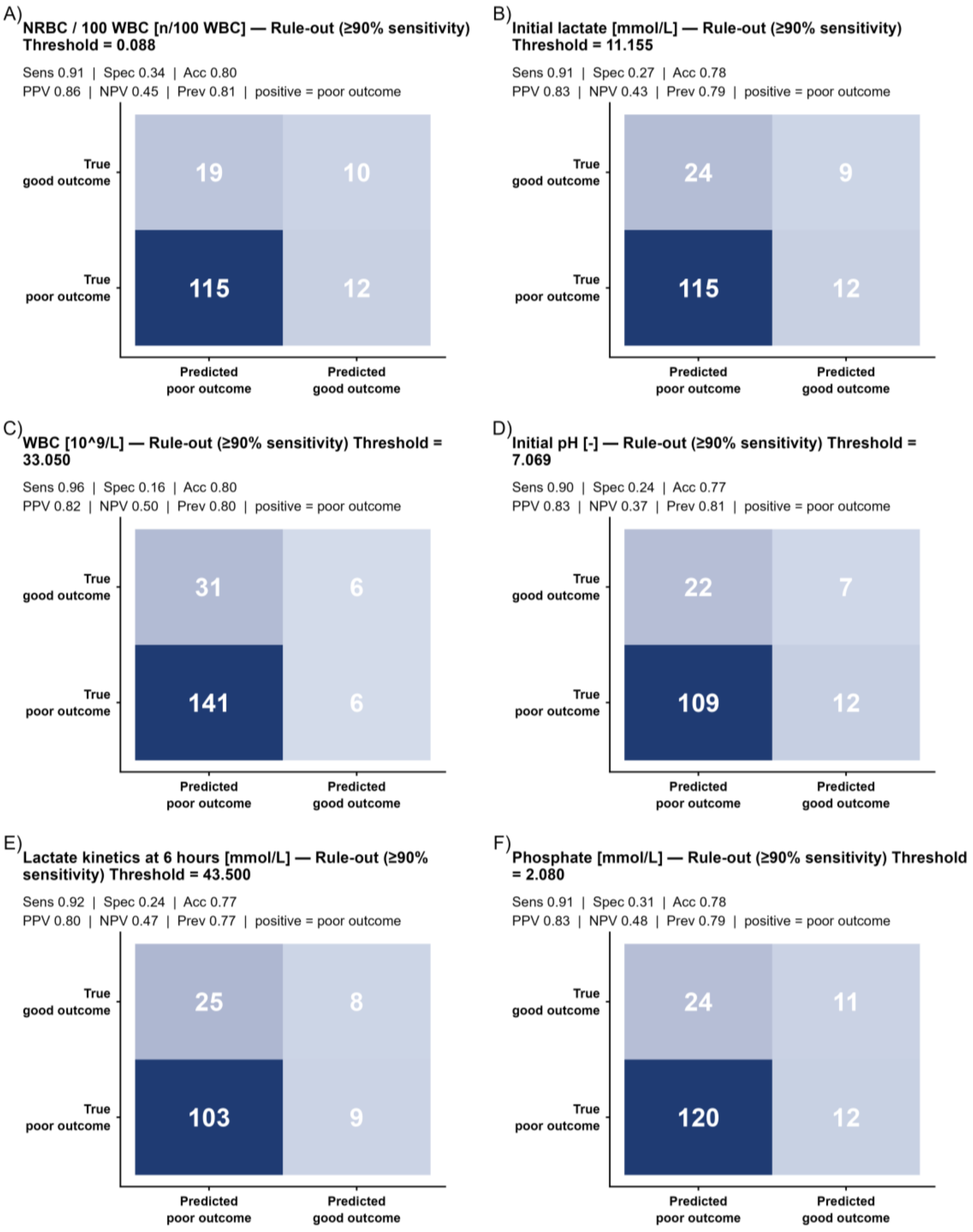

Supplemental Figure 3. Confusion matrices at rule-out thresholds (≥ 90 % sensitivity). *Each panel displays the confusion matrix for one laboratory marker at the rule-out threshold, defined by ≥ 90 % sensitivity. Poor outcome was defined as the positive condition. TP = true positives, FP = false positives, FN = false negatives, TN = true negatives, Sens = sensitivity, Spec = specificity, PPV = positive predictive value, NPV = negative predictive value, Acc = overall accuracy, Prev = prevalence of poor outcome.*

Supplemental Table 4. Univariable logistic regression models using continuous values for prediction of poor neurological outcome

Variable	OR (95% CI)	p-value	N	Poor outcomes	Good outcomes
NRBC / 100 WBC (per unit increase)	3.406 (0.931–12.463)	0.064	156	127	29
Lactate (per mmol/L increase)	1.184 (1.064–1.318)	0.002	160	127	33
pH (per unit increase)	0.018 (0.001–0.284)	0.004	150	121	29
Time to ECMO (per minute increase)	1.010 (0.991–1.029)	0.309	172	137	35

Supplemental Table 5. Univariable logistic regression models using thresholds for prediction of poor neurological outcome

Variable	Odds Ratio (95% CI)	p-value	N	Poor outcomes	Good outcomes
NRBC / 100 WBC ≥ 0.54	6.671 (1.514–29.391)	0.012	156	127	29
Lactate [mmol/L] ≥ 18.43	4.432 (1.276–15.395)	0.019	160	127	33
pH ≤ 6.72	2.489 (0.700–8.858)	0.159	150	121	29
Shockable rhythm	0.381 (0.169–0.862)	0.020	190	153	37

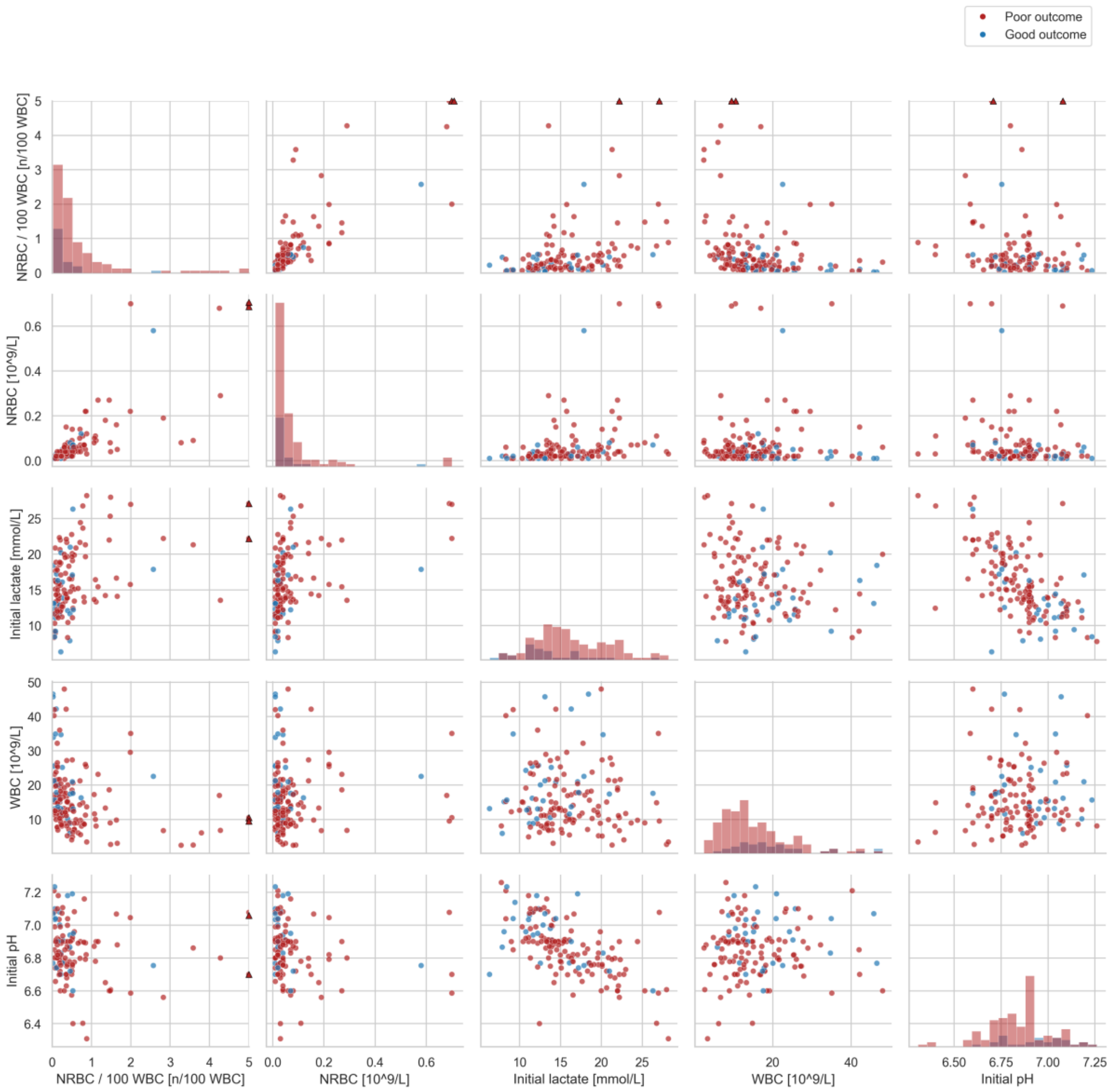

Supplemental Figure 4 *– Pairplot of Clinical Variables by Outcome: This figure shows pairwise relationships between the selected laboratory variables colored by patient outcome. Red indicates poor outcome, blue indicates good outcome. Each scatter and histogram visualizes the distribution and potential linear or nonlinear associations across all patients.*

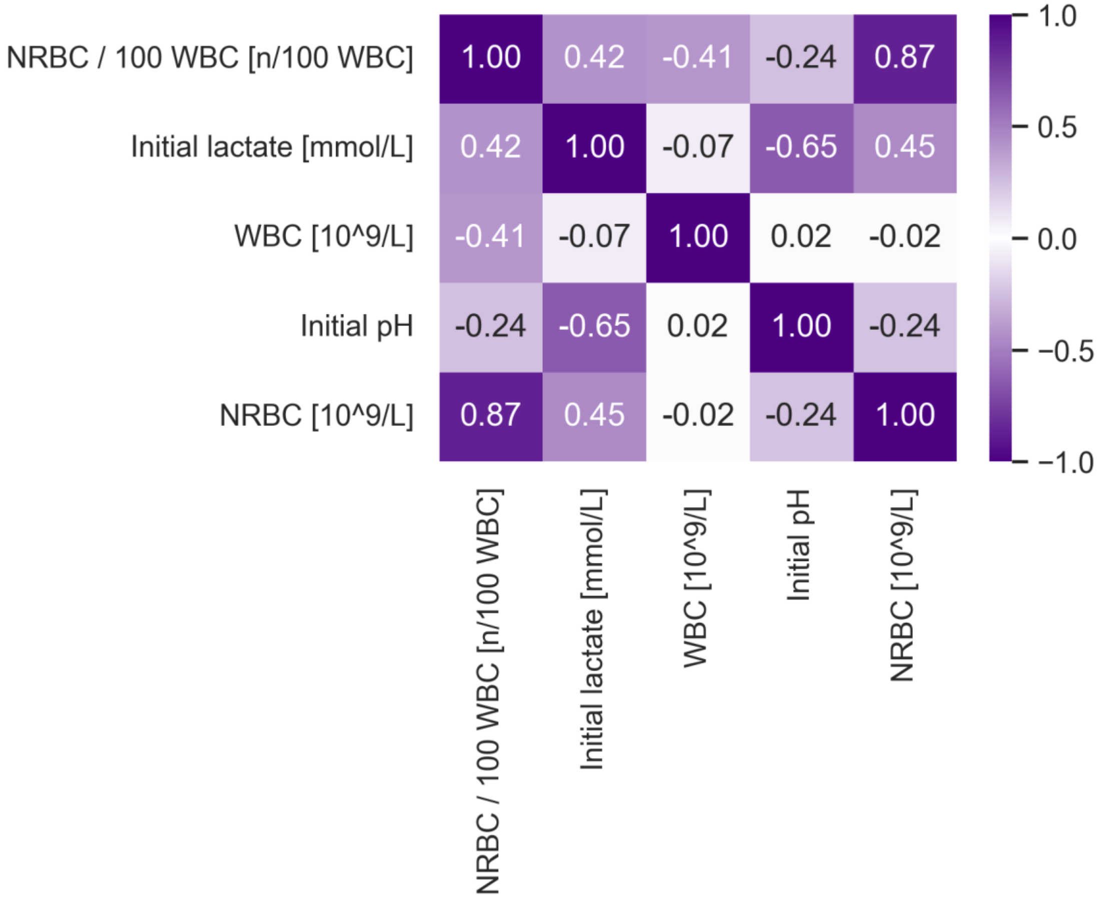

Supplemental Figure 5 – Spearman Correlation Heatmap (All Patients): *This heatmap displays the Spearman correlation coefficients among the same variables for the entire cohort. Purple shades indicate the strength and direction of linear correlations — darker tones represent stronger positive or negative relationships, while white indicates weak or no correlation.*

Supplemental Table 6. Overlap of NRBC per 100 WBC and NRBC absolute positivity with other laboratory predictors defined by study-specific cutoffs

Marker (positive by rule-in threshold)	NRBC / 100 WBC positive (n=44)	NRBC absolute positive (n=26)
WBC	56.8 % (n = 25)	26.9 % (n = 7)
Initial lactate	38.6 % (n = 17)	38.5 % (n = 10)
Initial pH	20.5 % (n = 9)	30.8 % (n = 8)
Phosphate	27.3 % (n = 12)	42.3 % (n = 11)
Lactate kinetics	31.8 % (n = 14)	42.3 % (n = 11)
NRBC / 100 WBC	100 % (n = 44)	88.5 % (n = 23)
NRBC absolute	52.3 % (n = 23)	100 % (n = 26)

N=number

Supplemental Table 7. Combined biomarker rule-in and rule-out performance

Marker combination	Rule	Sensitivity	Specificity	True poor outcome	False poor outcome	True good outcome	False good outcome	PPV	NPV	N
NRBC / 100 WBCs + Initial lactate	Rule-in	0.17	1.0	17	0	–	–	1.0	–	127
NRBC / 100 WBCs + Initial pH	Rule-in	0.09	1.0	9	0	–	–	1.0	–	120
NRBC / 100 WBCs + Initial lactate	Rule-out	0.96	0.08	–	–	2	4	–	0.33	127
NRBC / 100 WBCs + Initial pH	Rule-out	0.98	0.09	–	–	2	2	–	0.50	120

PPV = positive predictive value, NPV= negative predictive value

Supplemental Table 8. Patients fulfilling the nRBC rule-in criterion without metabolic marker positivity

Group	n	Poor outcome	Good outcome	Poor outcome (%)	Good outcome (%)
NRBC ≥0.54 but lactate <18.43 mmol/L	15	14	1	93.3%	6.7%
NRBC ≥0.54 but pH >6.72	22	21	1	95.5%	4.5%

Supplemental Table 9. Frequency of established lactate and pH thresholds among patients with good neurological outcome after ECPR (adjusted for missing data)

Threshold	True / False Poor Outcome
Lactate ≥ 14 mmol/L	80 / 11 (12.1%)
Lactate ≥ 15.1 mmol/L	68 / 10 (12.8%)
Lactate ≥ 20 mmol/L	27 / 3 (10.0%)
pH ≤ 6.8	48 / 8 (14.3%)

number (%),
